# TGF β1 and PDGF AA override Collagen type I inhibition of proliferation in human liver connective tissue cells

**DOI:** 10.1186/1471-230X-4-30

**Published:** 2004-12-03

**Authors:** Alvaro T Geremias, Marcelo A Carvalho, Radovan Borojevic, Alvaro NA Monteiro

**Affiliations:** 1 Departamento de Bioquímica, Instituto de Química, Universidade Federal do Rio de Janeiro, Rio de Janeiro 21949, Brazil; 2 Laboratório de Metabolismo Macromolecular Firmino Torres de Castro, Instituto de Biofísica Carlos Chagas Filho, Universidade Federal do Rio de Janeiro, Rio de Janeiro 21941, Brazil; 3 Departamento de Histologia e Embriologia, Instituto de Ciências Biomédicas, Universidade Federal do Rio de Janeiro, Rio de Janeiro 21941, Brazil

## Abstract

**Background:**

A marked expansion of the connective tissue population and an abnormal deposition of extracellular matrix proteins are hallmarks of chronic and acute injuries to liver tissue. Liver connective tissue cells, also called stellate cells, derived from fibrotic liver have been thoroughly characterized and correspond phenotypically to myofibroblasts. They are thought to derive from fat-storing Ito cells in the perisinusoidal space and acquire a contractile phenotype when activated by tissue injury. In the last few years it has become evident that several peptide growth factors such as PDGF AA and TGF-β are involved in the development of fibrosis by modulating myofibroblast proliferation and collagen secretion. The fact that during the development of chronic fibrosis there is concomitant deposition of collagen, a known inhibitory factor, and sustained cell proliferation, raises the possibility that stellate cells from chronic liver fibrosis patients fail to respond to normal physiologic controls.

**Methods:**

In this study we address whether cells from fibrotic liver patients respond to normal controls of proliferation. We compared cell proliferation of primary human liver connective tissue cells (LCTC) from patients with liver fibrosis and skin fibroblasts (SF) in the presence of collagens type I and IV; TGF-β, PDGF AA and combinations of collagen type I and TGF-β or PDGF AA.

**Results:**

Our results indicate that despite displaying normal contact and collagen-induced inhibition of proliferation LCTC respond more vigorously to lower concentrations of PDGF AA. In addition, we show that collagen type I synergizes with growth factors to promote mitogenesis of LCTC but not SF.

**Conclusions:**

The synergistic interaction of growth factors and extracellular matrix proteins may underlie the development of chronic liver fibrosis.

## Background

In normal liver, connective tissue cells are rare and mostly restricted to the periportal and pericentrovenular spaces and to the Glisson's capsule. A minor population of connective tissue cells is present inside the hepatic lobule, in the perisinusoidal space. They are known as hepatic stellate cells (HSC) and are considered to be one of the major contributors for fibrogenesis in liver [1]. Chronic and acute injuries to liver tissue induce a marked expansion of the connective tissue cells and concomitantly an abnormal deposition of extracellular matrix proteins [2]. Extensive studies of experimental models and humans have shown that these cells are of the myofibroblast lineage, characterized by the expression of smooth muscle α-actin [3-5]. In fact, there is now increasing evidence that are several populations of myofibroblasts in the diseased liver in addition to those derived from HSC [6,7]. The origin of these cells is still debated. In experimental models, it is considered that an acute liver injury is followed by activation and increase of stellate cells, while chronic injuries elicit activation of similar cells that can be either of lobular or periportal origin [7]. In humans, extensive primary periportal fibrosis such as schistosomal fibrosis was related to expansion of myofibroblasts originating from the portal vein wall [8], while in cirrhosis resident periportal connective tissue cells, as well as lobular stellate cells and pericentrovenular cells were reported to convert into myofibroblasts [3,9,10]. Independently of their origin, connective tissue cells of human fibrotic and cirrhotic liver tissues were shown to have homogeneous morphologic characteristics, as well as a specific growth patterns [11-15].

Recent studies on the mechanisms controlling connective tissue cell expansion and abnormal collagen deposition during liver fibrosis have shown that several peptide growth factors are involved in the development of fibrosis. Evidence from *in vivo *and *in vitro *studies indicated that TGF-β has a relevant role in the development of fibrosis, being a potent regulator of proliferation and of extracellular matrix protein synthesis [16-18]. TGF-β is highly expressed in fibrotic regions and overexpression of TGF-β in animal models results in fibrosis [18,19]. Conversely, TGF-β antagonists can prevent experimentally induced fibrosis [20-22]. PDGF AA was shown to have a pivotal role in controls of normal and pathologic proliferation of two myofibroblastic cell lineages: mesangial cells in kidney and alveolar smooth muscle cells in lungs [23,24].

On the other hand, it has also been demonstrated *in vitro *that the extracellular matrix can in its turn modulate cellular responses to peptide growth factors [25]. *In vitro*, fibroblast proliferation can be regulated by the presence of collagen type I. It has been suggested that collagen induces a quiescent state by decreasing the responsiveness to different growth factors [26,27]. An increase in cell density is also known to inhibit fibroblast proliferation *in vitro*. During the development of chronic fibrosis there is concomitant deposition of collagen and sustained cell proliferation, raising the possibility that liver connective tissue cells (LCTC) from chronic liver fibrosis patients fail to respond to normal physiologic controls.

In this study we addressed the question of whether cells from fibrotic liver patients respond to normal physiologic controls of proliferation. We compared the behavior of primary human LCTC from patients with liver fibrosis with skin fibroblasts (SF). We compared cell proliferation in the presence of collagen types I and IV, of TGF-β and PDGF AA, and in combinations of collagen type I and TGF-β or PDGF AA. Our results indicate that LCTC respond more vigorously to lower concentrations of peptide growth factors than SF. Interestingly, collagen type I synergizes with the growth factors tested in promoting mitogenesis of LCTC but not of SF. We believe that this mechanism may underlie the development of chronic liver fibrosis.

## Methods

### Cell lines

Liver tissue fragments and skin biopsies were obtained through collaboration with the University Hospital, Department of Surgery, Federal University of Rio de Janeiro (O.M. Vieira, M.D.). An informed consent was obtained from every patient, and the study was conducted in agreement with the ethical guidelines of the Federal University of Rio de Janeiro. Diagnoses were given by the pathological anatomy service of the hospital. All surgical liver biopsies were obtained for diagnostic purposes during spleno-renal or porto-caval anastomosis. Skin fibroblasts were obtained from the mid-abdominal incision performed for the surgery. We studied six primary normal SF lines and ten primary LCTC lines derived from patients with schistosomal fibrosis or alcoholic cirrhosis.

Biopsies were collected and brought to the laboratory in chilled Dulbecco's modified Eagle's medium (DMEM) (Sigma, St. Lois, MO) with 10% fresh human serum and 60 μg/ml of gentamicin (Schering, Rio de Janeiro, Brazil). They were cut into pieces of approximately 1 mm^3^, washed in fresh medium and plated, 6 or 9 pieces per 25 cm^2 ^flask. They were maintained in DMEM supplemented with 4 g/liter HEPES and 10% fresh human serum, at 37°C in a humid incubator with 5% CO_2_, 95% air. All the cultures used in this study were finite cell lines derived from the primary cultures described above, and were discarded before the 10th passage.

LCTC have been described in detail previously, and compared to skin fibroblasts and vascular smooth muscle cells [12,13,15]. They were characterized by electron microscopy, immunofluorescence, morphology and proliferation in culture and ability to contract a collagen matrix. These established primary cell cultures are homogeneous in terms of production of smooth muscle α-actin, fibronectin, collagen I and III, and elements of the basement membrane such as collagen IV and laminin [12,13,15]. Hence, they can be phenotypically characterized as myofibroblasts and different from fibroblasts or smooth muscle cells.

### Serum and growth factors

Fresh human citrated plasma was obtained from the hemotherapy service of the Hospital dos Servidores do Estado (Rio de Janeiro, Brazil). Plasma was coagulated with calcium and sera from several patients were pooled. Human TGF-β1 and human recombinant PDGF-AA were purchased from Sigma Chemical Company, St Louis, MO. TGF-β1 was activated by incubation in bovine serum albumin 1 mg/ml and 4 mM HCl.

### Preparation of collagen coated dishes

One hundred μl of solutions with varying concentrations of collagen type I (rat tail tendon collagen, prepared in our laboratory as previously described [13]) or collagen type IV (Sigma) were dispensed onto a 12-well or a 96-well plate and dried under a laminar flow for 24 hr at room temperature. This procedure allowed us to control for the exact amount of collagen added to each plate. The plates used were freshly prepared and were washed three times in serum free medium.

### Proliferation

Cell proliferation was assayed by cell counting and [H^3^]-thymidine incorporation. These methods correlated well with autoradiography results in previous studies [15]. For the experiments to assay contact inhibition of proliferation, approximately 1 × 10^3 ^cells/cm^2 ^were plated. Each day, during a period of 8 days, cells were trypsinized and counted in a hemocytometer and medium was changed every two days. To assay the proliferative response induced by growth factors and extracellular matrix proteins cells were trypsinized and plated at a concentration of 5 × 10^3 ^and allowed to adhere for 2 hr in DMEM with 10% serum. Then, cells were starved in serum-free medium for 18 hr to synchronize the cell population. When testing for growth factors effects, serum-free medium was replaced by medium containing 1% serum, supplemented with the growth factors and 0.5 μCi/ml [H^3^]-thymidine. When assessing the effect of extracellular matrix proteins, serum-free medium was replaced by medium containing 10% serum. Cells were collected 24 or 32 hr after the stimulus and lysed in 10 N NaOH. The trichloroacetic acid precipitable material was then spotted on a filter and counted on a scintillation counter.

### Kinetics of thymidine incorporation

Cells were plated at a concentration of 2.5 × 10^3 ^per well (0.5 cm^2^) in 96 well plates in DMEM with 10% serum. Cells were allowed to adhere and spread for two hr. Medium was then replaced with serum-free medium for 24 hr. Subsequently, cells were incubated in different media containing 1% serum and growth factors. [H^3^]-thymidine (1 μCi/ml) was added at 6, 24, 32 and 40 hr and incubated for 2 hr. Cells were harvested and processed as described above.

### Adhesion and recovery

To determine cell adhesion on different substrata plates were prepared with 0.3 mg/ml collagen type I or IV. Cells were plated on each substratum and the supernatant was removed at 0, 10, 20, 40 and 60 min and the cells remaining in suspension counted in a hemocytometer. To determine cell recovery, cells plated after 2 hr were trypsinized and counted.

### Statistical analysis

Difference between the means of various subgroups was assessed using the Mann-Whitney U-test.

## Results

Chronic liver fibrosis is characterized by abnormal proliferation of connective tissue cells. Therefore, we hypothesized that connective tissue cells from fibrotic lesions have lost normal proliferation controls. By using a series of primary connective tissue cell lines from skin and from fibrotic livers, we investigated several parameters of cell growth *in vitro *in order to identify potential mechanisms to explain the excessive proliferation of LCTC during fibrosis. We observed no differences among the cell lines obtained from patients with schistosomal fibrosis or alcoholic cirrhosis, and all the studied primary LCTC lines were included in a single experimental series. We established and characterized 16 primary human cell lines. The experiments performed were done at or before the tenth passage and every experiment was performed with at least two cell lines from each type (LCTC or SF) isolated from different individuals.

### Contact inhibition of proliferation

**Figure 1:**
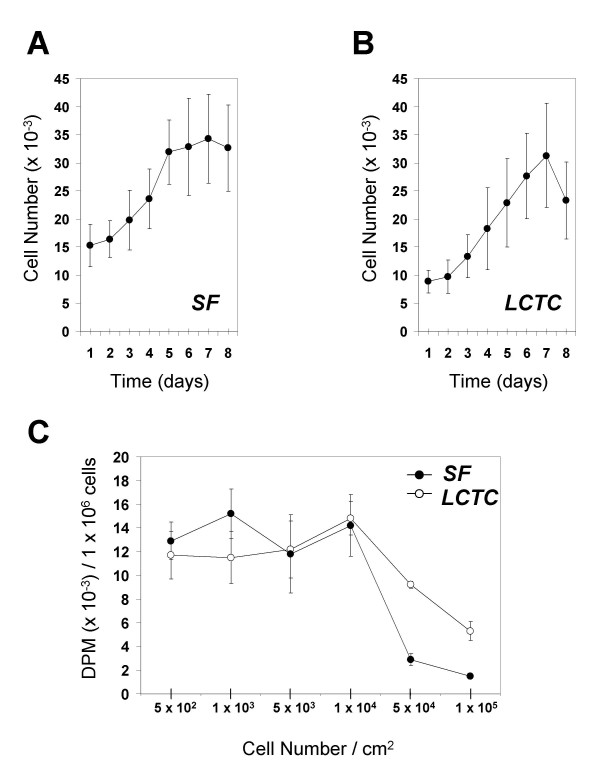
**Contact inhibition of proliferation. A. **Proliferation of normal human skin fibroblasts (SF) in vitro. **B. **Proliferation of human liver connective tissue cells (LCTC). Cells were plated at 1 × 10^3 ^cells/cm^2^. Each day, during a period of 8 days, cells were trypsinized and counted in a hemocytometer (*n *= 3). Medium containing 10% serum was replaced every two days. **C. **[H^3^]-Thymidine incorporation in SF and LCTC at increasing cell density (*n *= 4).

To assess whether connective tissue cells from fibrotic livers (LCTC) and normal skin fibroblasts (SF) responded to contact inhibition of proliferation, we measured cell proliferation during eight days. Both LCTC and SF showed a comparable pattern of growth and reached a plateau at a similar cell density (Figure 1A,1B). Reproducibly, LCTC declined in number after reaching a maximal density (Figure 1B, day 8). To confirm these results, we measured [H^3^]-thymidine incorporation in various cell densities (Figure 1C). Both LCTC and SF showed maximum [H^3^]-thymidine incorporation at the same cell density (1 × 10^4^/cm^2^) and a marked decline at higher densities. We also examined the response of both cell types to increasing concentrations of fresh human serum. Both SF and LCTC reached maximum proliferation, as measured by [H^3^]-thymidine incorporation at 20% serum (not shown). In conclusion, primary SF and LCTC were similarly responsive to normal contact inhibition of proliferation and serum concentration, although SF seemed to respond more efficiently to contact inhibition.

### The effects of extracellular matrix

**Figure 2:**
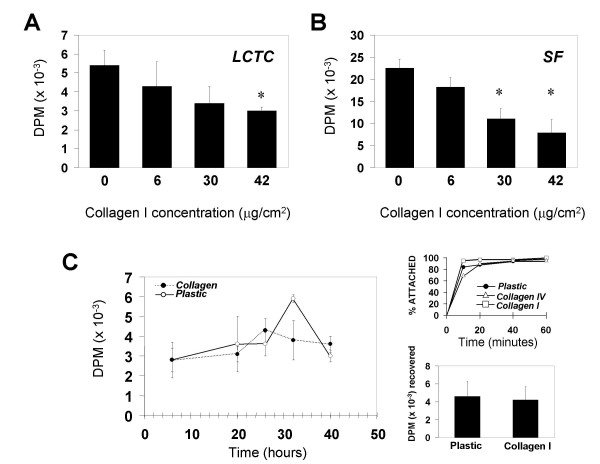
**Collagen-mediated inhibition of proliferation. A. **Cell proliferation of LCTC plated on increasing collagen concentration as measured by [H^3^]-Thymidine incorporation (*n *= 4). Note inhibition of incorporation in a dose-dependent manner. **B. **Cell proliferation of SF plated on increasing collagen concentration as measured by [H^3^]-Thymidine incorporation (*n *= 4). Note inhibition of incorporation in a dose-dependent manner. (*) denotes significant difference from control; *p *≤ *0.05*. **C. **Left panel. Kinetics of DNA synthesis of LCTC comparing growth on plastic (empty circles) and on collagen (filled circles) (*n *= 4). Right panel, top. Adhesion of cells plated on plastic (filled circles), collagen IV (empty triangles) and collagen I (empty squares) (*n *= 3). Right panel, bottom. Recovery of cells from plastic or collagen I (*n *= 4).

Despite the abnormal deposition of extracellular matrix in chronic fibrotic livers, LCTC continuously proliferate suggesting that these cells are not responsive to normal inhibition of proliferation by collagen I. To determine the influence of the extracellular matrix on LCTC, we analyzed DNA synthesis in the presence of collagen type I and type IV, two major components of extracellular matrix in fibrotic livers. Collagen type I inhibited DNA synthesis in a dose-dependent manner, as measured by [H^3^]-thymidine incorporation in LCTC and SF (Figure 2A,2B). In SF, [H^3^]-thymidine incorporation at the highest collagen concentration was approximately 30% of the control, suggesting that this cell type is more susceptible to collagen-mediated inhibition of proliferation. However, the level of the inhibition was variable among the studied patients, and a less marked reduction, (~50%) was observed in SF derived from some of the studied cases (not shown). We did not observe any change in cell morphology dependent upon the substrate.

The question of whether the difference observed between collagen-coated and plastic plates resulted from an alteration in kinetics of cell cycle progression or to an actual block in DNA synthesis was addressed in the following experiment. After serum-mediated activation of quiescent cells, the cultures were pulsed with [H^3^]-thymidine for 2 hr, at 6, 20, 26, 32 and 40 hr. [H^3^]-thymidine incorporation reached its peak at 32 hours after activation in control experiments (plated on plastic), and cells plated on collagen-coated plates did not show any delayed peak, demonstrating that the effect of collagen was not delaying the progression of cell cycle but blocking DNA synthesis (Figure 2C). These results indicate that LCTC are responsive to normal inhibition of proliferation mediated by collagen I.

Next, we asked whether collagen type IV could also influence the proliferation of the studied cells. Even at high concentrations (90 μg/cm^2^), collagen type IV did not induce any significant change on [H^3^]-thymidine incorporation in two LCTC cell lines, neither in two SF cell lines (not shown). Again, no difference in morphology was noted.

To rule out the possibility that the results were being masked by differential adhesion to collagen or differential cell recovery when measuring the [H^3^]-thymidine incorporation, we monitored cell adhesion on plastic, on collagen type I and on collagen type IV. Although by 10 min there were a significantly less cells attached on collagen IV, by 40 min the same number of cells had attached to the plates in all substrates (Figure 2C, right panel). These results rule out the possibility that lower [H^3^]-thymidine incorporation found in cells plated on collagen I was due to differential adhesion, since our experiments allowed 2 hr for attachment. We also counted cell numbers on the samples recovered for scintillation counting and found it to be comparable (Figure 2C, right panel), thus ruling out the possibility that the difference was due to differential recovery. Taken together these results indicate that LCTC are responsive to normal inhibition of proliferation mediated by collagen I in a manner similar to that of normal SF.

### Effects of PDGF AA and TGF-β on proliferation

**Figure 3:**
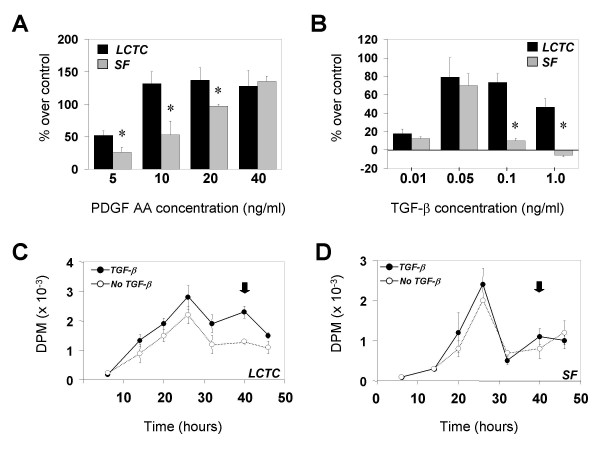
**Mitogenic effects of PDGF AA and TGF-β on SF and LCTC. A. **Effect of increasing PDGF AA concentrations on growth of LCTC (black bars) and SF (gray bars) (*n *= 4). **B. **Effect of increasing concentrations of TGF-β on growth of LCTC (black bars) and SF (gray bars) (*n *= 4). (*) denotes significant difference between SF and LCTC; *p *≤ *0.05*. **C. **Kinetics of DNA synthesis in LCTC as measured by [H^3^]-thymidine incorporation in the presence (filled circles) and absence (empty circles) of TGF-β (*n *= 4). Note second delayed peak of incorporation at 40 hr (black arrow). **D. **Kinetics of DNA synthesis in SF as measured by [H^3^]-thymidine incorporation in the presence (filled circles) and absence (empty circles) of TGF-β (*n *= 4). Note the absence of the second delayed peak of incorporation at 40 hr (black arrow).

To investigate the effects of PDGF AA we starved cells for 18 hr and stimulated them with increasing concentrations of PDGF AA. As shown in Figure 3A, LCTC were more sensitive to lower concentrations of the growth factor (5, 10 and 20 ng/ml), reaching a peak at 10 ng/ml. Although reaching the same level of stimulation at 40 ng/ml, SF were less sensitive to PDGF AA at lower concentrations. PDGF AA did not affect timing of cell cycle progression, since cells in the presence and in the absence of PDGF AA displayed a peak of [H^3^]-thymidine incorporation at 26 hr after release in medium containing serum and factors (results not shown). In conclusion, LCTC appear to be more sensitive to lower concentrations of PDGF AA.

To test the responsiveness of LCTC and SF to TGF-β, cells were starved overnight and stimulated with increasing concentrations of acid-activated TGF-β. In Figure 3B we note that at lower concentrations, stimulation of LCTC and SF is comparable. However, higher concentrations of TGF-β (0.1 ng/ml) seemed to be less effective in promoting mitogenesis of SF. Interestingly, at the highest concentration (1 ng/ml) TGF-β was inhibitory to SF but still highly stimulatory for LCTC. When we analyzed the kinetics of thymidine incorporation during TGF-β treatment (Figure 3C,3D) we observed a second delayed peak of DNA synthesis at 40 hr (Figure 3C and 3D, arrow). In smooth muscle cells this second delayed peak has been shown to be due to a PDGF AA autocrine loop [28]. While in LCTC the second peak was 85% of the first peak, in SF it was slightly under 50%. These results indicate that TGF-β can induce a prolonged and strong induction of DNA synthesis in LCTC even at concentrations that are inhibitory for SF. In this set of experiments we were able to compare matched LCTC and SF from the same patient. Our results indicate that the observed differences between LCTC and SF are not due to individual variation or genetic background.

### Synergy of extracellular matrix and growth factors

**Figure 4:**
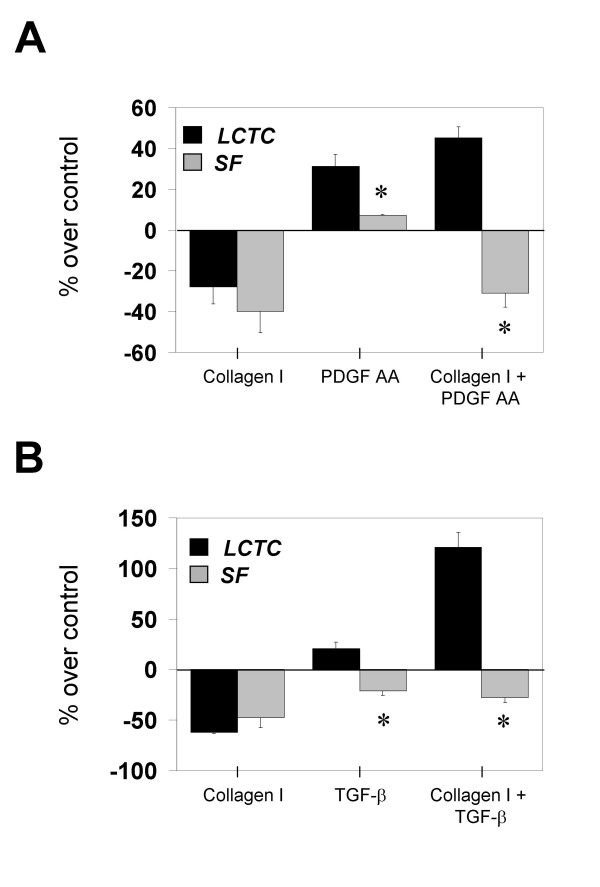
**Interaction between peptide growth factors and extracellular matrix. A. **Effects of PDGF AA on the growth of LCTC (black bars) and SF (gray bars) plated on collagen or plastic (*n *= 4). **B. **Effects of TGF-β on the growth of LCTC (black bars) and SF (gray bars) plated on collagen or plastic (*n *= 3). (*) denotes significant difference between SF and LCTC; *p *≤ *0.05*.

Next, we assessed the interplay between growth factors and the extracellular matrix on the stimulation of proliferation. We compared proliferation of cells cultured on collagen film using the highest inhibitory concentration (42 μg/cm^2^; Figure 2) and cultured on plastic. Initially, we treated cells with 10 ng/ml of PDGF AA in the presence and absence of collagen after 24 hr (Figure 4A). Both LCTC and SF were efficiently inhibited by collagen, in accordance with the experiments in Figure 2A and 2B, performed with different cell lines. In the absence of collagen, PDGF AA stimulated proliferation comparable to that obtained in Figure 3A and 3B. These conditions provide an internal control to distinguish effects that are cell type-specific from individual variations. Surprisingly, PDGF AA was able to override the collagen-mediated inhibition only in LCTC and not in SF.

Next, cells were stimulated with TGF-β (Figure 4B) and proliferation measured. Since there was a possibility that the autocrine loop could induce a late response, we assessed [H^3^]-thymidine incorporation at 40 hr. (Figure 4B). Collagen inhibited these cell lines as in previous experiments with LCTC and SF cell lines. TGF-β alone had little effect on proliferation. However, similarly to the experiment with PDGF AA, TGF-β was not only able to override the collagen-mediated inhibition but showed a synergistic effect only in LCTC. SF were not able to escape inhibition mediated by collagen.

## Discussion

Injuries to liver tissue usually involve transient or long-term development of fibrosis. In acute injuries, fibrotic tissue is frequently reabsorbed and normal tissue architecture is restored. In cases when the primary agent or the secondary pathogenic mechanisms are persistent, the fibrotic reaction can be perpetuated causing a severe impairment of organ function [1]. It is therefore important to identify the factors that are involved in this perpetuation in order to devise more efficient preventive measures and therapies. However, there is a dearth of knowledge about the behavior of the human LCTC isolated from fibrotic livers. In fact, most studies done to date used LCTC cells from experimental models such as rats and mice and the few studies dealing with myofibroblasts from human liver derived the cells from normal tissue.

In order to evaluate the hypothesis that LCTC from fibrotic livers had lost control of proliferation we investigated their growth in tissue culture. Contact inhibition of proliferation is a characteristic of normal cells and is lost in neoplastic cell lines. Our results showed that LCTC respond normally to contact inhibition of proliferation. Collagen type I, which is the predominant extracellular matrix protein deposited during hepatic fibrogenesis [2], has been shown to be a potent inhibitor of mesenchymal cell proliferation [26,29,30]. Since the perpetuated proliferation of LCTC is a hallmark of chronic liver diseases, it was conceivable that these cells had lost normal collagen-mediated inhibition. However, our results indicate that LCTC derived from fibrotic livers are responsive to collagen I to an extent comparable to normal skin fibroblasts. In addition, collagen IV a major component of basement membranes did not affect the growth of either cell type studied.

While recent evidence from rat vascular smooth muscle cells indicates that PDGF AA promotes only protein synthesis without activation of DNA synthesis [31], we demonstrate that PDGF AA is active as a mitogen for human LCTC. These results are in accordance with previous data using connective tissue cells derived from normal liver [32]. Interestingly, the same authors have found that in fibrotic livers there is marked increase in the expression of both PDGFα receptor and its ligand PDGF AA [33]. Taken together, these results strengthen the notion that PDGF-AA is an important mediator of connective tissue expansion during liver fibrosis.

Although generally agreed to contribute to the fibrogenic process by upregulating expression of genes encoding extracellular matrix proteins, a role for TGF-β in promoting mitogenesis has emerged in the last few years. TGF-β displayed a bi-phasic curve with low concentrations stimulating and higher doses inhibiting the proliferation of SF. On the other hand, TGF-β was a potent mitogenic stimulus for LCTC even at high concentrations. Interestingly, it has been shown that TGF-β also stimulates an additional delayed growth response mediated by an autocrine loop of PDGF AA [28], which we observed only in LCTC.

One surprising outcome of this study was that not only the peptide growth factors tested were able to override the collagen-mediated inhibition, but that they were also able to synergize with the extracellular matrix. Since the collagen we used was a crude preparation from rat tail tendon we cannot rule out the possibility that the observed effect may depend on other associated proteins that are known to be accessory factors in promoting more efficient ligand-binding to the receptor. These results reinforce the notion that connective tissue cells can be therapeutically controlled using a strategy to inhibit the action of peptide growth factors.

It is well known that continuous presence of the injury agent determines the evolution of the disease, favoring its progression and perpetuation (establishment of a permanent scar tissue). On the other hand, it is largely unclear which are the other physiologic determinants for disease evolution. Genetic background is known to influence the development of hypertrophic cheloid skin scars as well as the outcome of schistosomal liver fibrosis [34]. In experiments in which we were able to use matched LCTC and SF from the same individual our results suggest that tissue origin was consistently more important than the genetic background, since LCTC behaved differently from the same-patient's SF but similarly to LCTC from different individuals.

## Conclusions

The results obtained in this study using human primary liver connective tissue cells suggest a plausible scenario for the development of liver fibrosis. Stellate quiescent cells are triggered to proliferate by high molecular weight serum factors [15] and may be subjected to a positive autocrine and paracrine feedback loop of growth factor stimulation. Whereas under normal conditions connective tissue cells are kept under strict check by the extracellular matrix, PDGF AA and TGF-β are capable of not only overriding the inhibitory effect caused by collagen type I on LCTC but also synergize to provide a stronger stimulus for proliferation. This interplay of extracellular signals may underlie the development of irreversible liver fibrosis.

## List of abbreviations

HEPES: N-[2-Hydroxyethyl] piperazine-N'-[2-ethanesulfonic acid]; HSC: hepatic stellate cells; LCTC: liver connective tissue cells; SF: skin fibroblasts; PDGF: platelet-derived growth factor; TGF-β: transforming growth factor-β.

## Competing interests

The author(s) declare that they have no competing interests.

## Authors' contributions

AG established primary cell line cultures, carried out the cell biological studies, participated in designing and interpreting the experiments. MC provided technical support for the cell cultures and participated in the writing of the manuscript. RB participated in the design of the study and in the interpretation of the results. AM established primary cultures, conceived of the study, and participated in its design and coordination. All authors read and approved the final manuscript.

## Pre-publication history

The pre-publication history for this paper can be accessed here:


